# Oscillation Kinetics of Post-illumination Increase in Chl Fluorescence in Cyanobacterium *Synechocystis* PCC 6803

**DOI:** 10.3389/fpls.2016.00108

**Published:** 2016-02-15

**Authors:** Min Xu, Jing Lv, Pengcheng Fu, Hualing Mi

**Affiliations:** ^1^National Key Laboratory of Plant Molecular Genetics, Institute of Plant Physiology and Ecology, Shanghai Institutes for Biological Sciences, Chines Academy of SciencesShanghai, China; ^2^Renewable Energy Research Center, China University of PetroleumBeijing, China

**Keywords:** post-illumination increase in Chl fluorescence, *Synechocystis* sp. PCC 6803, NAD(P)H dehydrogenase, cyclic electron flow around photosystem I, plastoquinone

## Abstract

After termination of longer-illumination (more than 30 s), the wild type of *Synechocystis* PCC 6803 showed the oscillation kinetics of post-illumination increase in Chl fluorescence: a fast phase followed by one or two slow phases. Unlike the wild type, *ndh*-B defective mutant M55 did not show any post-illumination increase under the same conditions, indicating that not only the fast phase, but also the slow phases were related to the NDH-mediated cyclic electron flow around photosystem I (PS I) to plastoquinone (PQ). The fast phase was stimulated by dark incubation or in the presence of Calvin cycle inhibitor, iodoacetamide (IA) or cyclic photophosphorylation cofactor, phenazine methosulphate (PMS), implying the redox changes of PQ by electrons generated at PS I reduced side, probably NAD(P)H or ferredoxin (Fd). In contrast, the slow phases disappeared after dark starvation or in the presence of IA or PMS, and reappeared by longer re-illumination, suggesting that they are related to the redox changes of PQ by the electrons from the photoreductants produced in carbon assimilation process. Both the fast phase and slow phases were stimulated at high temperature and the slow phase was promoted by response to high concentration of NaCl. The mutant M55 without both phases could not survive under the stressed conditions.

## Introduction

Similar to eukaryotic phototrophs, cyanobacteria carry out oxygen photosynthesis with two distinguishable photosystems, photosystem I (PS I) and photosystem II (PSII; Scherer, 1990). Light energy is converted into biochemical energy by driven transfer of electron from water to terminal electron acceptor NADP^+^. Thus, the linear electron flow produces ATP by a proton gradient across the thylakoid membrane, coupling the formation of NADPH. In addition to the linear electron flow, there is a cyclic electron flow around PS I, which converts light to electrochemical potential for synthesis of ATP. Cyclic electron flow around PS I is considered physiological important not only for its providing extra ATP for carbon assimilation (Schurman et al., 1972; Mills et al., 1978; Slovacek et al., 1978), but also for its adjusting the production ratio of ATP to NADPH for developmental stages (Hatch, 1987), environmental stress (Mi et al., 2001; Wang et al., 2006), and physiological requirements (Xu et al., 2014). Since lacking organelles such as chloroplasts and mitochondria, respiratory electron transport chain of cyanobacteria couples with photosynthetic intersystem chain by sharing some components (Jones and Myers, 1963). The respiratory electron transport which can produce ATP in darkness and remove oxygen plays an important role in nitrogen fixation (Scherer et al., 1988).

In cyanobacteria, respiration and cyclic electron flow are mediated by cyanobacterial type-1 NAD(P)H dehydrogenase (NDH-1) complexes which functions in a variety of bioenergetic reactions (Mi et al., 1992) and CO_2_ uptake (Ogawa, 1991). NDH-1 consists of 17 subunits at least, among those, NdhA–NdhK are homologous to those of *Escherichia coli* complex I (Friedrich and Scheide, 2000), NdhL–NdhQ specially exist in cyanobacteria, identified by functional proteomics approach (Prommeenate et al., 2004; Battchikova et al., 2005) or by purification (Nowaczyk et al., 2011). NDH-1 from cyanobacteria is speculated to possess an oxygenic photosynthesis-specific (OPS) domain (Birungi et al., 2010) comprised of NdhL–NdhO identified in *Synechocystis* 6803 (Prommeenate et al., 2004; Battchikova et al., 2005). Several NDH subunits function in stabilization of NDH-1. NdhQ is also essential for stabilization of the large complex of NDH-1 (Zhao et al., 2015). NdhP is involved in the respiratory and cyclic electron flow (Schwarz et al., 2013) and is essential to stabilize the NDH-1L complex (Zhang et al., 2014). NdhS as a novel subunit of NDH-1 participates in the cyclic electron flow in *Arabidopsis* (Yamamoto et al., 2011) and also in cyanobacteria (Battchikova et al., 2012) that it serves as the docking site for Fd, accepting electrons from Fd in chloroplasts (Yamamoto and Shikanai, 2013).

Post-illumination increase in Chl fluorescence has been found in C4 plant (Asada et al., 1993), C3 plant (Mano et al., 1995), and cyanobacteria (Mi et al., 1995). The phenomenon was explained as the reduction of plastoquinone (PQ) by the electrons from photoreductants accumulated in the stroma or cytosol during illumination, and may reflect cyclic electron transport around PS I mediated by NDH-1 (Mi et al., 1995) and by plastid NADH dehydrogenase-like complex (NDH; Sazanov et al., 1998; Shikanai et al., 1998). It could be used as a convenient way to study the activity of cyclic electron flow around PS I *in vivo*.

In this work, we found that after termination of illumination, the wild type of *Synechocystis* sp. PCC 6803 (hereafter *Synechocystis* PCC 6803) showed the oscillation kinetics of post-illumination increase in Chl fluorescence. The oscillation kinetics was further investigated under different conditions combined with the ndhB defective mutant (M55) and inhibitors. The results showed that the oscillation is suggested to be related to the changes in the redox state of PQ by electrons from various photoreductants and responses to environmental stresses.

## Materials and Methods

### Culture of Cyanobacteria

*Synechocystis* PCC 6803 and its *ndh*B defective mutant, M55 (Ogawa, 1991), were cultured in the BG-11 medium buffered with Tris-HCl (5 mM, pH 8.0; Allen, 1968) at 27°C under fluorescent lamps at 60 μmol m^-2^ s^-1^ bubbling with 2% CO_2_ in air. According to the experiments, the cells at logarithmic growth stage were used for measurements.

### Measurement of the Kinetics of Changes in Chl Fluorescence

The redox change of PQ was monitored by Chl fluorescence, using a PAM Chlorophyll Fluorometer (Walz, Effeltrich, Germany) with an emitter–detector–cuvetter assembly (ED-101 US). Details for fluorometer setup were described previously (Schreiber et al., 1994, 1995). The cell suspension was pipetted in a cuvetter with a thermostat. Cells were exposed to the actinic light (AL, 3 Wm^-2^) for 30 s. Then AL was turned off and the transient increase kinetics in chlorophyll fluorescence was detected. Each sample was adapted in the dark for 2 min prior to measurement. As for treatment with the cyclic photophosphorylation cofactor, 100 μM of phenazine methosulphate (PMS) was added to the sample before measurement; and with the Calvin cycle inhibitor, iodoacetamide (IA) was pre-incubated with the sample at low concentration (40 μM) and high concentration (1 mM). As for dark starvation, cells of *Synechocystis* PCC 6803 were pre-treated in dark for 24–32 h before measurement. Every experiment was repeated three times at least.

### Oxygen Exchange

The evolution of oxygen under the white light (150 W m^-2^) was determined in a reaction mixture that contained the mid-logarithmic cell of *Synechocystis* PCC 6803 in the cultural BG11 medium at 5 μg Chl ml^-1^ with 10 mM NaHCO_3_ with a Clark-type oxygen electron. The reaction mixture kept at indicated temperatures as shown in **Figure 3A** with a thermostat.

## Results

### Oscillation Kinetics of Post-illumination Increase in Chl Fluorescence

**FIGURE 1:**
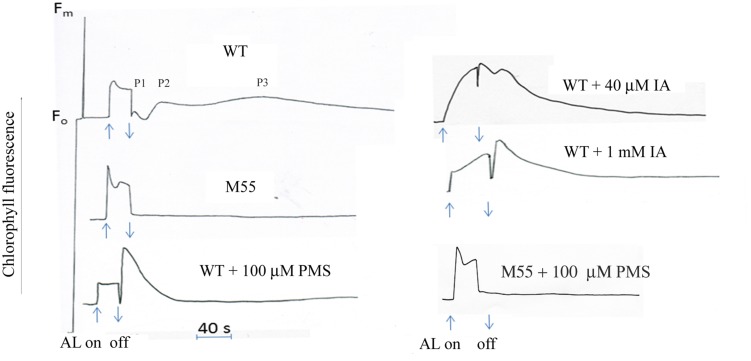
**Kinetics of post-illumination increase in Chl fluorescence.** The Top curve shows a typical trace of chlorophyll fluorescence in wild type (WT) *Synechocystis* PCC 6803. The cell at mid-logarithmic stage (OD730 ≈ 0.5) was pipetted in a cuvetter with a thermostat. After the cells were exposed to the AL (3 Wm^-2^) for 30 s, the transient increase kinetics in chlorophyll fluorescence was detected. The wild type showed the oscillation kinetics of post-illumination increase in Chl fluorescence: a fast phase, peak1 (P1) followed by one or two slow phase peak2 (P2) or peak3 (P3). M55, NdhB defective mutant; PMS, phenazine methosulphate; IA, iodoacetamide. Each sample was adapted in the dark for 2 min prior to the measurement. The chemicals were, respectively, added to the sample before adaptation in the dark. Every experiment was independently repeated three times at least.

The post-illumination increase in Chl fluorescence is used to reflect the activity of cyclic electron flow around PS I in cyanobacteria and high plants. The kinetics usually is monophasic under certain conditions in the previous studies (Deng et al., 2003). Here we found that after termination of the longer-illumination(more than 30 s) of red light (3 W m^-2^), the wild type of *Synechocystis* PCC 6803 showed a oscillation of post-illumination increase in Chl fluorescence: a fast phase, peak1 (P1) followed by one or two slow phases peak2 (P2) or peak3 (P3) (**Figure 1**). In contrast, *ndh*B defective mutant M55 did not show any post-illumination increase under the same conditions (**Figure 1**), indicating that not only fast phase, but also slow phases related to the reduction of PQ by the NDH-mediated cyclic electron flow. To know what kinds of components contribute to the different phases, we checked effect of a cyclic electron cofactor and a Calvin cycle inhibitor as follows.

### Effect of Inhibitors on the Kinetics of Post-illumination Increase in Chl Fluorescence

Phenazine methosulphate is a well-known artificial cyclic photophosphorylation cofactor, which bypasses electrons from PS I acceptor side to the P700 (Trebst, 1974). Addition of PMS significantly stimulated the increase of fast phase, but suppressed the slow phases of post-illumination increase in Chl fluorescence in the wild type, but not in M55 (**Figure 1**), suggesting that the fast phase is caused by the electron donation from simple substrates, probably NADPH, and slow phase is caused by electron donation from the substrates produced in the process of carbon assimilation, and NDH-1 is involved in these processes. To test the possibility, the effect of IA, an inhibitor of Calvin cycle which blocks linear electron flow but stimulates cyclic electron flow around PS I in spinach chloroplasts (Joliot and Alric, 2013), on post-illumination increase in Chl fluorescence was checked. Both the P1 and P2 were significantly stimulated but the P3 disappeared at the presence of low concentration of IA (40 μM; **Figure 1**). Moreover, high concentration of IA (1 mM) stimulated the fast phase but suppressed the slow phase (**Figure 1**). The results indicate that fast phase reflects the redox change of PQ by electrons from PS I reduced side, probably NADPH or Fd and the slow phases by those from complicate components synthesized through the Calvin cycle.

### Effect of Dark Starvation and Re-illumination on the Oscillation Kinetics of Post-illumination Increase in Chl Fluorescence

**FIGURE 2:**
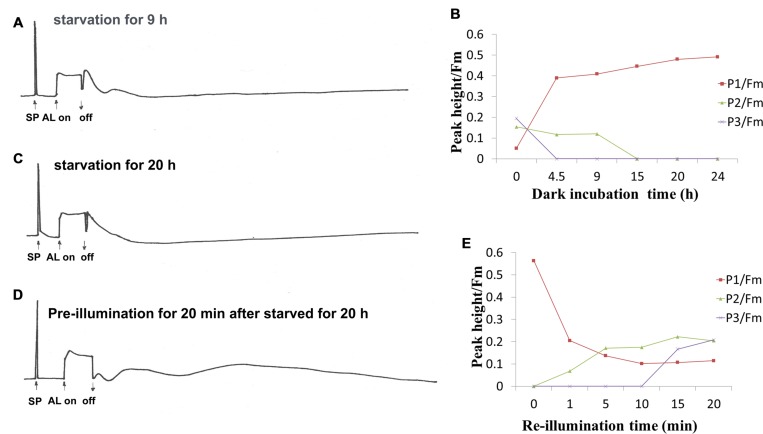
**Effect of dark starvation and re-illumination on the oscillation kinetics of post-illumination increase in Chl fluorescence in WT *Synechocystis* PCC 6803.** After the cell at mid-logarithmic stage (OD730 ≈ 0.5) was incubated in the dark for indicated times, the kinetics was measured. **(A)** Dark-starvation for 9 h; **(C)** dark-starvation for 20 h; **(B)** the time course of changes in peak1 (P1), peak2 (P2), and peak3 (P3) during dark adaption; **(D)** the kinetics of the 20 h-dark-starved cells after re-illumination for 20 min; **(E)** the time course of the changes in P1, P2, and P3 after re-illumination at different times. The measurement conditions were the same as in **Figure 1**. Every experiment was independently repeated three times at least.

Given that the redox state of PQ is also affected by donation of electrons from respiratory substrates, the effect of dark incubation on the oscillation kinetics was carried out. It has been known that dark starvation for 24–32 h could cause a shortage of respiratory substrates, but the cells still retained their photosynthetic capacity in the *Synechocystis* cells (Mi et al., 1994). With dark incubation, P1 increased, while P2 and P3 decreased (**Figures 2A,B**). P1 reached to the maximal value after 24 h, P3 and P2 disappeared after 4.5 and 15 h dark incubation, respectively (**Figures 2B,C**). When the 25-h dark-starved cells were re-illuminated for 20 min, the level of P1 fell close to that before the dark incubation while P2 and P3 rose to the level before dark incubation (**Figures 2D,E**). These results suggest that the electrons from PS I reduced side contributes to P1 and respiratory substrates such as glucose and other intermediate such as triose phosphate might affect the P2 and P3.

### Effect of Temperature on Kinetics of Post-illumination Increase in Chl Fluorescence

**FIGURE 3:**
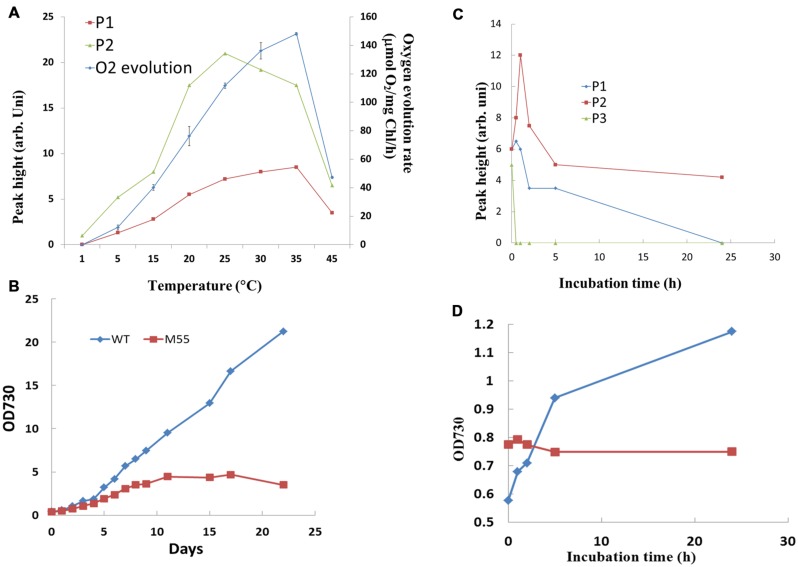
**Effect of temperature **(A)** and high concentration of NaCl **(C)** on the oscillation kinetics of post-illumination increase in Chl fluorescence in WT *Synechocystis* PCC 6803.** Comparison of the effect of high temperature (34°C) **(B)** and 0.8 M NaCl **(D)** on the growth between WT and M55. The measurement conditions were the same as in **Figure 1**. Every experiment was independently repeated three times at least.

To further understand the relation of redox changes of PQ with photosynthetic activity, the response of the oscillation kinetics to different temperatures was measured. As shown in **Figure 3A**, the peak height of both the fast and slow phases and rate of oxygen evolution increased with the rise of temperature and reached to the maximal values until 25–35°C, respectively. The three parameters decreased dramatically when the temperature reached 45°C. These results indicate that both the fast phase and slow phase of post-illumination increase in Chl fluorescence display a similar temperature response with the photosynthetic electron transport and other reactions consuming energy, which are all enhanced with the rise of temperature within the limits of physiological temperature. To know the physiological significance of the kinetics, the growth of wild type and NdhB defective mutant M55 without both kineticses has been compared at 34°C. As shown in **Figure 3B**, the growth of M55 was much slower as compared to the wild type and could not further grow after 10 days. The result suggests that the enhancement of both fast and slow phases related with the cyclic electron flow around PS I mediated by NDH-1 is required for the heat tolerance for the *Synechocystis* PCC 6803.

### Effect of NaCl Stress on the Oscillation Kinetics of Post-illumination Increase in Chl Fluorescence

It has been demonstrated that NDH-1 dependent cyclic electron flow is essential for short-term adaptation of *Synechocystis* to salt shock (Tanaka et al., 1997). The response of the oscillation kinetics to salt stress was investigated. As shown in **Figure 3C**, the P2 was significantly increased, reached to the maximum at 1h and then gradually decreased. P1 was only slightly stimulated, but P3 was decreased after incubation of the cells in 0.8 M NaCl. By comparison with the wild type, M55 could not grow in the culture medium contained 0.8 M NaCl (**Figure 3D**), suggesting that the stimulated P2 related with NDH-pathway is essential for salt stress response and tolerance in *Synechocystis* PCC 6803.

## Discussion

Post-illumination increase in Chl fluorescence is widely used for investigation of the activity of cyclic electron flow around PS I mediated by NDH-1, which reflects the reduction of PQ pool by the electrons from the photoreductants generated in PS I during illumination in cyanobacteria (Mi et al., 1995; Deng et al., 2003) and high plants (Sazanov et al., 1998; Shikanai et al., 1998). This work studied the oscillation kinetics of the post-illumination increase in Chl fluorescence for first time. The significant enhancement of the fast phase by adding the cofactor (PMS) of cyclic electron flow (**Figure 1**) or by dark incubation (**Figure 2**), indicating that the fast phase of the kinetics reflects the donation of electrons to PQ pool from the photoreductants generated in PS I, such as reduced fd, NADPH, that is the short-cut cyclic electron flow around PS I. Moreover, the increase of the fast phase of kinetics but suppression of slow phase in presence of high concentration of IA (**Figure 1**), an inhibitor of Calvin cycle, as well as the re-appearance of the slow phases after re-illumination the starved cells for 20 min (**Figure 2**) indicate that the slow phase may reflect the donation of electrons from the photoreductants generated in process of carbon assimilation, probably triose phosphates, glucose. It has been proposed the existence of a large pool of electron donors when illuminating the dark starvation cells of *Synechocystis* PCC 6803 by AL continues for more than 10 s, as analyzed by the complementary areas of the re-oxidation curve of P700 by far-red light after illumination with AL (Mi et al., 1994). Thus, the slow phase kinetics of the post-illumination increase in Chl fluorescence probably represents the reduction of the PQ pool by such a large pool of electrons accumulated in stroma or cytosol during illumination. Both the fast phase and slow phases were not detectable in NdhB defective mutant, M55, even in the presence of cyclic co-factor PMS (**Figure 1**), suggesting that NDH-1 is involved in the mediation of electron donation to the PQ pool from different substrates. The previous study has shown that the activity of both the respiratory flow and cyclic electron flow is light-dependent (Mi et al., 2001), therefore, the light inducible slow phases might be reflected the redox states of PQ pool by the electron donation from the respiratory flow as well as the cyclic electron flow.

As one of photoprotective pathways, cyclic electron flow around PS I mediated by NDH has been found to be enhanced under stressed conditions, such as high temperature (Wang et al., 2006), strong light (Mi et al., 2001), salt shock (Tanaka et al., 1997). In our results, the increase of both the fast and slow phases as well as the enhanced activity of photosynthetic oxygen evolution at higher physiological temperature (**Figure 3A**) suggests that the cyclic electron flow has close relationship with activity of CO_2_ assimilation under stressed condition. It has been indicated that cyclic electron flow around PS I plays an important role in providing ATP for carbon assimilation (Schurman et al., 1972; Slovacek and Hind, 1981). Actually, the ATP supply could not always satisfy the demand of the plant during the later grain filling stage (Shen, 1994). Evidence to support this suggestion is that the enhancement of cyclic electron flow around PS I by treatment with low concentration of NaHSO_3_ enables plants or cyanobacteria to generate sufficient proton gradient across the thylakoid membrane and ATP (Wu et al., 2011; Rolfe et al., 2012), thereby increased the biomass of cyanobacterium *Synechocystis* PCC 6803 (Wang et al., 2003). The research of mutants impaired in both NDH- and PGR5-pathways have indicated that cyclic electron flow around PS I is required for the efficient photosynthesis (Munekage et al., 2002). Thus, the growth of M55 was severely suppressed at 34°C when the activity of cyclic electron flow around PS I mediated by NDH-1 is impaired by knocking out the *NdhB* gene (**Figure 3B**). Salinity stress is one of the most serious factors limiting the productivity of plants. One of strategies to adapt salinity condition is to exclude sodium ion from cytosol by Na^+^/H^+^ antiporter Na^+^ ATPase in plants (Blumwald and Poole, 1987; Apse et al., 1999) or in cyanobacteria (Kaku et al., 1999). The exclusion of Na^+^ is an energy consuming process, while cyclic electron flow around PS I provides extra ATP in this demand. Based on the stimulation of the donation of electrons from the cytosol to P700^+^ via NDH-1, it has been suggested that the cyclic electron flow mediated by NDH-1 might play an important role in the exclusion of Na^+^ ions from salt-shocked cells of *Synechocystis* PCC 6803 (Tanaka et al., 1997). The significantly stimulated slow phase, P2, at high salt stress (**Figure 3C**) indicates the large electron pool contributed to the cyclic electron flow may function for providing ATP for salinity tolerance. Impairment of both the fast and slow phases in M55 increased the sensitivity to the high salt stressed conditions (**Figure 3D**), supporting the above mention.

## Conclusion

The studying the oscillation kinetics of the post-illumination increase in Chl fluorescence enables us to distinguish the different phases as the indicators for electron donations to the PQ pool from different photoreductants via NDH-mediated cyclic electron flow around PS I, and to use the kinetics to analyze the physiological responses to environmental stresses in cyanobacteria.

## Author Contributions

All authors listed, have made substantial, direct and intellectual contribution to the work, and approved it for publication. MX performed the research work and manuscript revision. JL helped performing experiments. PF discussed for the study. HM designed the research and wrote the paper.

## Conflict of Interest Statement

The authors declare that the research was conducted in the absence of any commercial or financial relationships that could be construed as a potential conflict of interest.

## References

[B1] Allen M. M. (1968). Simple conditions for growth of unicellular blue-green algae on plates. *J. Phycol.* 4 1–4. 10.1111/j.1529-8817.1968.tb04667.x 27067764

[B2] Apse M. P. Aharon G. S. Snedden W. A. Blumwald E. (1999). Salt tolerance conferred by overexpression of a vacuolar Na^+^/H^+^ antiport in *Arabidopsis*. *Science* 285 1256–1258. 10.1126/science.285.5431.1256 10455050

[B3] Asada K. Heber U. Schreiber U. (1993). Electron flow to the intersystem chain from stromal components and cyclic electron flow in maize chloroplasts, as detected in intact leaves by monitoring redox change of P700 and chlorophyll fluorescence. *Plant Cell Physiol.* 34 39–50.

[B4] Battchikova N. Wei L. Du L. Bersanini L. Aro E.-M. Ma W. (2012). Identification of novel Ssl0352 protein (NdhS), essential for efficient operation of cyclic electron transport around photosystem I, in NADPH: plastoquinone oxidoreductase (NDH-1) complexes of *Synechocystis* sp. PCC 6803. (vol 286, pg 36992, 2011). *J. Biol. Chem.* 287 8660–8660. 10.1074/jbc.M111.263780 PMC3196108 21880717

[B5] Battchikova N. Zhang P. P. Rudd S. Ogawa T. Aro E. M. (2005). Identification of NdhL and Ssl1690 (NdhO) in NDH-1L, and NDH-1M complexes of *Synechocystis* sp PCC 6803. *J. Biol. Chem.* 280 2587–2595. 10.1074/jbc.M410914200 15548534

[B6] Birungi M. Folea M. Battchikova N. Xu M. Mi H. Ogawa T. (2010). Possibilities of subunit localization with fluorescent protein tags and electron microscopy examplified by a cyanobacterial NDH-1 study. *Biochim. Biophys. Acta* 1797 1681–1686. 10.1016/j.bbabio.2010.06.004 20547137

[B7] Blumwald E. Poole R. J. (1987). Salt tolerance in suspension-cultures of sugar-beet – Induction of NA^+^/H^+^ antiport activity at the tonoplast by growth in salt. *Plant Physiol.* 83 884–887. 10.1104/pp.83.4.884 16665356 PMC1056467

[B8] Deng Y. Ye J. Y. Mi H. L. (2003). Effects of low CO2 on NAD(P)H dehydrogenase, a mediator of cyclic electron transport around photosystem I in the cyanobacterium *Synechocystis* PCC6803. *Plant Cell Physiol.* 44 534–540. 10.1093/pcp/pcg067 12773640

[B9] Friedrich T. Scheide D. (2000). The respiratory complex I of bacteria, archaea and eukarya and its module common with membrane-bound multisubunit hydrogenases. *FEBS Lett.* 479 1–5. 10.1016/S0014-5793(00)01867-6 10940377

[B10] Hatch M. D. (1987). C-4 photosynthesis: a unique blend of modified biochemistry, anatomy and ultrastructure. *Biochim. Biophys. Acta* 895 81–106. 10.1016/S0304-4173(87)80009-5

[B11] Joliot P. Alric J. (2013). Inhibition of CO2 fixation by iodoacetamide stimulates cyclic electron flow and non-photochemical quenching upon far-red illumination. *Photosyn. Res.* 115 55–63. 10.1007/s11120-013-9826-1 23625532

[B12] Jones L. W. Myers J. (1963). A common link between photosynthesis and respiration in a blue-green alga. *Nature* 199 670–672. 10.1038/199670a0 14074553

[B13] Kaku N. Hibino T. Tanaka Y. Takabe T. Nakamura T. Takabe T. (1999). Expression of nhaAv gene encoding Na^+^/H^+^ antiporter from *Vibrio alginolyticus* in a freshwater cyanobacterium *Synechococcus* sp PCC 7942 confers lithium tolerance, but not sodium tolerance. *Plant Cell Physiol.* 40 557–564. 10.1093/oxfordjournals.pcp.a029577

[B14] Mano J. Miyake C. Schreiber U. Asada K. (1995). Photoactivation of the electron flow from NADPH to plastoquinone in spinach chloroplasts. *Plant Cell Physiol.* 36 1589–1598.

[B15] Mi H. Endo T. Schreiber U. Ogawa T. Asada K. (1994). NAD(P)H Dehydrogenase-dependent cyclic electron flow around Photosystem-I in the cyanobacterium *Synechocystis* PCC-6803: a study of dark-starved cells and spheroplasts. *Plant Cell Physiol.* 35 163–173.

[B16] Mi H. L. Deng Y. Tanaka Y. Hibino T. Takabe T. (2001). Photo-induction of an NADPH dehydrogenase which functions as a mediator of electron transport to the intersystem chain in the cyanobacterium *Synechocystis* PCC6803. *Photosyn. Res.* 70 167–173. 10.1023/A:1017946524199 16228350

[B17] Mi H. L. Endo T. Ogawa T. Asada K. (1995). Thylakoid membrane-bound, NADPH-specific pyridine-nucleotide dehydrogenase complex mediates cyclic electron-transport in the cyanobacterium *Synechocystis* sp PCC-68038. *Plant Cell Physiol.* 36 661–668.

[B18] Mi H. L. Endo T. Schreiber U. Ogawa T. Asada K. (1992). Electron donation from cyclic and respiratory flows to the photosynthetic intersystem chain is mediated by pyridine-nucleotide dehydrogenase in the cyanobacterium *Synechocystis* PCC-6803. *Plant Cell Physiol.* 33 1233–1237.

[B19] Mills J. D. Slovacek R. E. Hind G. (1978). Cyclic electron-transport in isolated intact chloroplasts – Further-studies with antimycin. *Biochim. Biophys. Acta* 504 298–309. 10.1016/0005-2728(78)90178-0 718878

[B20] Munekage Y. Hojo M. Endo T. Shikanai T. (2002). *Arabidopsis* pgr5 is defective in cyclic electron flow around photosystem I. *Plant Cell Physiol.* 43 S23–S23. 10.1016/s0092-8674(02)00867-x 12176323

[B21] Nowaczyk M. M. Wulfhorst H. Ryan C. M. Souda P. Zhang H. Cramer W. A. (2011). NdhP and NdhQ: two novel small subunits of the cyanobacterial NDH-1 complex. *Biochemistry* 50 1121–1124. 10.1021/bi102044b 21244052 PMC3040284

[B22] Ogawa T. (1991). A gene homologous to the subunit-2 gene of NADH dehydrogenase is essential to inorganic carbon transport of *Synechocystis* PCC6803. *Proc. Natl. Acad. Sci. U.S.A.* 88 4275–4279. 10.1073/pnas.88.10.4275 1903537 PMC51641

[B23] Prommeenate P. Lennon A. M. Markert C. Hippler M. Nixon P. J. (2004). Subunit composition of NDH-1 complexes of *Synechocystis* sp PCC 6803 – Identification of two new ndh gene products with nuclear-encoded homologues in the chloroplast Ndh complex. *J. Biol. Chem.* 279 28165–28173. 10.1074/jbc.M401107200 15102833

[B24] Rolfe M. D. Ocone A. Stapleton M. R. Hall S. Trotter E. W. Poole R. K. (2012). Systems analysis of transcription factor activities in environments with stable and dynamic oxygen concentrations. *Open Biol.* 2 120091 10.1098/rsob.120091 PMC3411108 22870390

[B25] Sazanov L. A. Burrows P. A. Nixon P. J. (1998). The plastid ndh genes code for an NADH-specific dehydrogenase: isolation of a complex I analogue from pea thylakoid membranes. *Proc. Natl. Acad. Sci. U.S.A.* 95 1319–1324. 10.1073/pnas.95.3.1319 9448329 PMC18756

[B26] Scherer S. (1990). do photosynthetic and respiratory electron-transport chains share redox proteins. *Trends Biochem. Sci.* 15 458–462. 10.1016/0968-0004(90)90296-N 1963954

[B27] Scherer S. Almon H. Boger P. (1988). Interaction of photosynthesis, respiration and nitrogen-fixation in cyanobacteria. *Photosyn. Res.* 15 95–114. 10.1007/BF00035255 24430856

[B28] Schreiber U. Bilger W. Neubauer C. (1994). “Chlorophyll fluorescence as a non-intrusive indicator for rapid assessment of *in vivo* photosynthesis,” in *Ecophysiology of Photosynthesis*, eds Schulze E.-D. Caldwell M. M. (Berlin: Springer-Verlag), 49–70.

[B29] Schreiber U. Hormann H. Neubauer C. Klughammer C. (1995). Assessment of photosystem II photochemical quantum yield by chlorophyll fluorescence quenching analysis. *Aust. J. Plant Physiol.* 22 209–220. 10.1071/PP9950209

[B30] Schurman P. Buchanan B. B. Arnon D. I. (1972). Role of cyclic photophosphorylation in photosynthetic carbon-dioxide assimilation by isolated chloroplasts. *Biochim. Biophys. Acta* 267 111–124. 10.1016/0005-2728(72)90143-0 4401676

[B31] Schwarz D. Schubert H. Georg J. Hess W. R. Hagemann M. (2013). The gene sml0013 of *Synechocystis* species strain PCC 6803 encodes for a novel subunit of the NAD(P)H oxidoreductase or complex i that is ubiquitously distributed among Cyanobacteria. *Plant Physiol.* 163 1191–1202. 10.1104/pp.113.224287 24089436 PMC3813643

[B32] Shen Y. K. (1994). Dynamic approaches to the mechanism of photosynthesis. *Photosynth. Res.* 39 3–13. 10.1007/BF00027138 24310996

[B33] Shikanai T. Endo T. Hashimoto T. Yamada Y. Asada K. Yokota A. (1998). Directed disruption of the tobacco ndhB gene impairs cyclic electron flow around photosystem I. *Proc. Natl. Acad. Sci. U.S.A.* 95 9705–9709. 10.1073/pnas.95.16.9705 9689145 PMC21403

[B34] Slovacek R. E. Hind G. (1981). Correlation between photosynthesis and the transthylakoid proton gradient. *Biochim. Biophys. Acta* 635 393–404. 10.1016/0005-2728(81)90037-2 7236671

[B35] Slovacek R. E. Mills J. D. Hind G. (1978). Function of cyclic electron-transport in photosynthesis. *FEBS Lett.* 87 73–76. 10.1016/0014-5793(78)80136-7

[B36] Tanaka Y. Katada S. Ishikawa H. Ogawa T. Takabe T. (1997). Electron flow front NAD(P)H dehydrogenase to photosystem I is required for adaptation to salt shock in the Cyanobacterium *Synechocystis* sp. PCC 6803. *Plant Cell Physiol.* 38 1311–1318.

[B37] Trebst A. (1974). Energy conservation in photosynthetic electron-transport of chloroplasts. *Annu. Rev. Plant Physiol. Plant Mol. Biol.* 25 423–458. 10.1146/annurev.pp.25.060174.002231

[B38] Wang H.-W. Mi H. Ye J.-Y. Deng Y. Shen Y.-K. (2003). Low concentrations of NaHSO3 increase cyclic photophosphorylation and photosynthesis in cyanobacterium *Synechocystis* PCC6803. *Photosynth. Res.* 75 151–159. 10.1023/A:1022813402265 16245085

[B39] Wang P. Duan W. Takabayashi A. Endo T. Shikanai T. Ye J. Y. (2006). Chloroplastic NAD(P)H dehydrogenase in tobacco leaves functions in alleviation of oxidative damage caused by temperature stress. *Plant Physiol.* 141 465–474. 10.1104/pp.105.070490 16428601 PMC1475475

[B40] Wu Y. Zheng F. Ma W. Han Z. Gu Q. Shen Y. (2011). Regulation of NAD(P)H dehydrogenase-dependent cyclic electron transport around PSI by NaHSO(3) at low concentrations in tobacco chloroplasts. *Plant Cell Physiol.* 52 1734–1743. 10.1093/pcp/pcr109 21828103

[B41] Xu M. Shi N. Li Q. Mi H. (2014). An active supercomplex of NADPH dehydrogenase mediated cyclic electron flow around Photosystem I from the panicle chloroplast of *Oryza sativa*. *Acta Biochim. Biophys. Sin.* 46 757–765. 10.1093/abbs/gmu064 25074414

[B42] Yamamoto H. Peng L. Fukao Y. Shikanai T. (2011). An Src homology 3 domain-like fold protein forms a ferredoxin binding site for the chloroplast NADH dehydrogenase-like complex in *Arabidopsis*. *Plant Cell* 23 1480–1493. 10.1105/tpc.110.080291 21505067 PMC3101538

[B43] Yamamoto H. Shikanai T. (2013). In planta mutagenesis of Src homology 3 domain-like fold of NdhS, a ferredoxin-binding subunit of the chloroplast NADH dehydrogenase-like complex in *Arabidopsis*: a conserved Arg-193 plays a critical role in ferredoxin binding. *J. Biol. Chem.* 288 36328–36337. 10.1074/jbc.M113.511584 24225949 PMC3868747

[B44] Zhang J. Gao F. Zhao J. Ogawa T. Wang Q. Ma W. (2014). NdhP is an exclusive subunit of large complex of NADPH dehydrogenase essential to stabilize the complex in *Synechocystis* sp strain PCC 6803. *J. Biol. Chem.* 289 18770–18781. 10.1074/jbc.M114.553404 24847053 PMC4081920

[B45] Zhao J. Rong W. Gao F. Ogawa T. Ma W. (2015). Subunit Q is required to stabilize the large complex of NADPH dehydrogenase in *Synechocystis* sp strain PCC 6803. *Plant Physiol.* 168 443–451. 10.1104/pp.15.00503 25873552 PMC4453799

